# Quantitative Analysis of Terpenic Compounds in Microsamples of Resins by Capillary Liquid Chromatography

**DOI:** 10.3390/molecules24224068

**Published:** 2019-11-10

**Authors:** H. D. Ponce-Rodríguez, R. Herráez-Hernández, J. Verdú-Andrés, P. Campíns-Falcó

**Affiliations:** 1 MINTOTA Research Group, Department of Analytical Chemistry, Faculty of Chemistry, University of Valencia, Dr Moliner 50, 46100 Burjassot, Valencia, Spain; henrypon@alumni.uv.es (H.D.P.-R.); pilar.campins@uv.es (P.C.-F.); 2 Department of Chemical Control, Faculty of Chemistry and Pharmacy, National Autonomous University of Honduras, Ciudad Universitaria, 11101 Tegucigalpa, Honduras

**Keywords:** resins, limonene, triterpenes, microsamples, capillary liquid chromatography (Cap-LC)

## Abstract

A method has been developed for the separation and quantification of terpenic compounds typically used as markers in the chemical characterization of resins based on capillary liquid chromatography coupled to UV detection. The sample treatment, separation and detection conditions have been optimized in order to analyze compounds of different polarities and volatilities in a single chromatographic run. The monoterpene limonene and the triterpenes lupeol, lupenone, β-amyrin, and α-amyrin have been selected as model compounds. The proposed method provides linear responses and precision (expressed as relative standard deviations) of 0.6% to 17%, within the 0.5–10.0 µg mL^−1^ concentration interval; the limits of detection (LODs) and quantification (LOQs) were 0.1–0.25 µg mL^−1^ and 0.4–0.8 µg mL^−1^, respectively. The method has been applied to the quantification of the target compounds in microsamples. The reliability of the proposed conditions has been tested by analyzing three resins, white copal, copal in tears, and ocote tree resin. Percentages of the triterpenes in the range 0.010% to 0.16% were measured using sample amounts of 10–15 mg, whereas the most abundant compound limonene (≥0.93%) could be determined using 1 mg portions of the resins. The proposed method can be considered complementary to existing protocols aimed at establishing the chemical fingerprint of these kinds of samples.

## 1. Introduction

Natural resins are plant secretions formed by complex mixtures of organic molecules, being terpenoids the predominant components. The number and proportion of these substances highly depend on the botanical origin and age of the resins. For example, in resins derived from plants of the genera *Burseraceae*, commonly referred to as copal, monoterpenic compounds such as pinene and limonene are the most abundant compounds in the volatile fraction, whereas triterpenoids such as lupine compounds, α-amyrin, and β-amyrin are predominant in the non-volatile fraction [1]. Resins have important applications in the paint and cosmetic industries [1,2]. Very recently, some of their constituents have attracted the attention of researchers because of their pharmacological effects as anti-inflammatory, antipruritic, anti-fungal and others [2,3,4,5,6]. Besides their industrial applications, resins have been used from ancient times for a variety of purposes including religious ceremonies and decoration of artworks. For this reason, over the past years, the analysis of resins has attracted interest in the characterization of archaeological objects [7,8,9].

Different approaches have been described for the chemical analysis of resins in archeological items using gas chromatography (GC) coupled to mass spectrometry (MS) [7,9,10], liquid chromatography (LC) coupled to MS [10] or UV detection [9,11,12] and thin layer chromatography (TLC) [11]. Most of those studies were aimed at differentiating the samples according to their botanical origin through the comparison of the chromatographic profiles of the extracts obtained from the samples (chemical fingerprinting), often in combination with chemometric tools [8,10]. Interestingly, none of those methods reported the quantitative composition of the target compounds. This can be most probably explained by the lack of reliable quantitative methods that can be applied to microsamples, as the low amount of sample available is a major limitation in such studies. The quantitative composition of resins could be used not only to discriminate resins by their botanical origin but also to explore the age and storage conditions of the samples [1,6]. Thus, methods that can be used to provide a better knowledge of the amounts of (at least) the major components of resins are still needed [13].

Because of the bioactive properties of some triterpenes such as lupeol and amyrins, different methods have been recently proposed for their quantification in different plant materials [5,14,15,16,17]. The amount of sample in those studies was not limited, and therefore, the required sensitivity for quantification could be achieved after exhaustive sample treatments of large amounts of the samples, including multiple extractions, purification, solvent evaporation, and redissolution. Very recently, the quantification of the triterpenes lupeol, α-amyrin and β-amyrin in copal resins used in folk ceremonies was described using LC and UV detection, although the analytical performance of the method applied was not reported [12]; moreover, due to the large amount of resin needed (0.5 g), the method might be unsuitable for the analysis of microsamples.

On the other hand, several difficulties arise when analyzing resins by chromatographic methods. First, the samples contain a large number of compounds with very different chemical properties. Some of the most abundant high molecular triterpenes are highly apolar (octanol–water partition coefficients, K_ow_ > 10^9.0^). Therefore, their separation under typical reversed-phase conditions is difficult because the choice of the mobile phase is rather limited [14]. When using absorbance detection, the lack of chromophores may be also a limitation, especially in the analysis of microsamples. As regards GC-based methods, most assays require a derivatization step before GC analysis, especially if low-volatile high-molecular triterpenes are going to be analyzed [7,9]. Because of the complexity of the samples, most assays have been focused only on one family of compounds, typically the triterpene fraction. Specific assays have also been developed to the characterization of volatile components of resins using GC [6]. Alternatively, different portions of the sample extract are analyzed under two or more different chromatographic conditions to obtain more exhaustive sample characterization [10,14].

**Figure 1:**
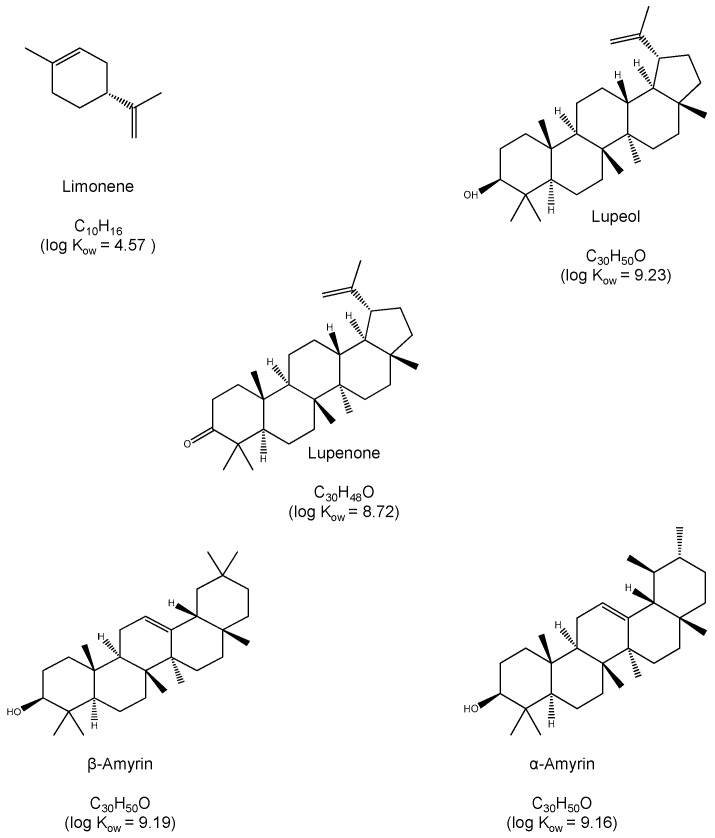
Chemical structures and log K_ow_ values of the tested compounds.

In this work, we describe a method for the quantification of representative components of resins, both volatile and non-volatile using capillary chromatography. The method takes advantage of the high sensitivity attainable with miniaturized LC systems, which make them better suited for the analysis of microsamples [18,19]. The volatile monoterpene limonene and the high molecular triterpenes lupeol, lupenone, α-amyrin and β-amyrin have been selected as model compounds. Their structure and octanol-water partition coefficients are shown in Figure 1. The analytical performance of the proposed method has been tested. Examples of application to real samples are presented.

## 2. Results

### 2.1. Chromatographic Conditions

Initially, different acetonitrile–water mixtures were tested in order to optimize the separation and detection of the target compounds. In this study, the percentage of acetonitrile ranged from 60% to 95%; standard solutions of the analytes (10 µg mL^−1^) prepared in methanol were used, and the injection volume was 5 µL.

**Figure 2:**
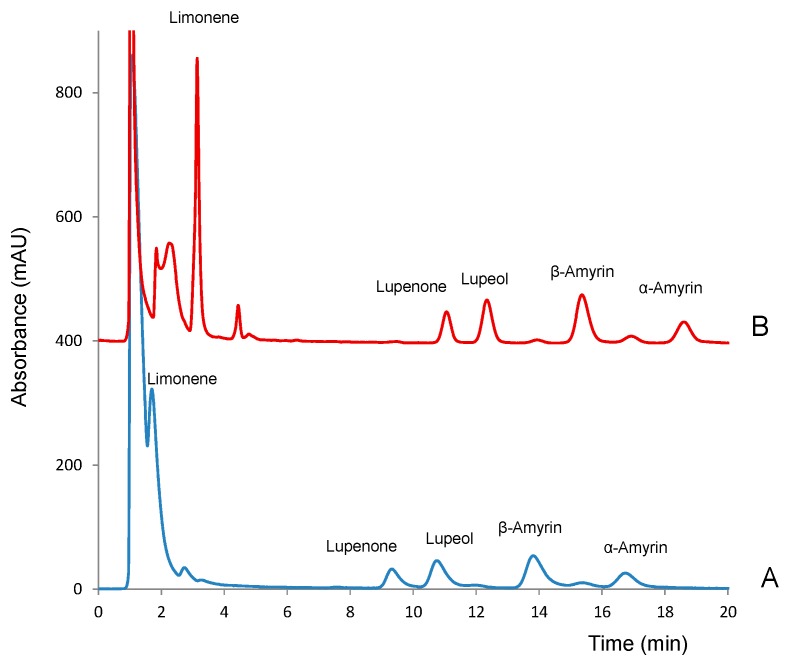
Obtained for standard solutions of the analytes (10 µg mL^−1^) in methanol injected (**A**) directly and (**B**) after loading 5 µL of water in the injection loop. Sample volume, 5 µL; eluent, 100% acetonitrile; detection wavelength, 200 nm.

As expected, mobile phases with high contents of acetonitrile (>70%) were necessary for the analytes to be eluted at reasonable run times (<40 min). It must be noted that all the analytes presented decreasing absorbances within the 190–210 nm range and nearly null absorbance at higher wavelengths. Thus, 200 nm was selected as the working wavelength. Under most of the elution conditions assayed suitable separation of the analytes was obtained except for limonene. The isolation of this compound was particularly difficult due to the presence of an intense peak corresponding to the injection solvent (methanol). Because of its high intensity, such peak partially overlapped with that of limonene. The resolution between the two peaks could be improved by using a gradient elution program but at the expense of the total run time. For the rest of the compounds, a good resolution was obtained even with a mobile phase of 100% acetonitrile; with this eluent, the chromatographic run time was <20 min, as shown in Figure 2A. Besides the peaks of the solvent and analytes, two minor peaks were detected at 12.1 min and 15.3 min; those peaks were identified as impurities of β-amyrin. Tetrahydrofuran was also tested as it has an elution strength higher than that of acetonitrile. However, due to its significant absorbance at wavelengths <212 nm, the background noise at the wavelength necessary to detect the analytes was unacceptable. Therefore, this solvent was no longer used.

As an attempt to reduce the solvent peak and to improve the resolution of limonene, standard solutions of the analytes were prepared using different methanol-water mixtures as solvent, 0.1:9.9, 1:9 and 9:1 (*v*/*v*) [14]. Ideally, samples should be injected in an injection solvent with elution strength similar to or lower than that of the mobile phase. However, the presence of water in the processed solutions resulted in a decrement of the peaks areas of some of the analytes, especially α-amyrin. This suggested that at the working concentration the analytes were not completely dissolved in methanol–water, which is consistent with their high Kow values (see Figure 1). As an alternative, we tested if the introduction of an aliquot of water in the injection capillary before loading the sample could prevent peak broadening at the entrance of the chromatographic column. Variable volumes of water in the 5–25 µL range were loaded in the injection loop, before loading the samples (5 µL), and the chromatograms were compared with those observed for the same solution directly injected (Figure 2A). The introduction of water into the injection capillary had a strong effect on the retention times of the analytes, as well as on peak shapes. As observed in Figure 2B, which shows the chromatogram obtained after the successive introduction of 5 µL of water and 5 µL of the working solution into the injection loop all the analytes eluted about 1.5 min later. This was particularly positive for the measurement of limonene, as it was completely separated from the solvent peak. The presence of water had also a positive effect on the peak shapes of the other analytes. Increasing the amount of water up to 25 µL did not modify substantially the chromatographic registers. Finally, the effect of the sample volume was evaluated with the range 5–25 µL. The absolute peak areas increased as the volume of the sample increased. However, the increment of the sample volume also resulted in wider peaks. As a result, the separation between lupeol and lupenone was unsuitable (data not shown).

Based on the above results, the successive injection into the loop of 5 µL of water and 5 µL of the working solution was selected as the best option. As a compromise between resolution and chromatographic run time, a mobile phase of acetonitrile:water 85:15 (*v*/*v*) was selected for further work.

### 2.2. Method Validation

To study the analytical performance of the proposed method, working solutions of the target compounds at concentrations in the range 0.25–10 µg mL^−1^ were analyzed, and the linearity, limits of detection (LODs), limits of quantification (LOQs), accuracy and precision were studied [20]. The results obtained are summarized in Table 1.

As observed from Table 1, for all the compounds tested the peak areas showed a linear relationship with the concentration up to 10.0 µg mL^−1^, with R^2^ coefficients ranging from 0.994 to 0.997 (*n* = 15). In order to check the accuracy, the corresponding calibration equations were used to establish the concentration of the analytes in solutions containing mixtures of the tested analytes at low-intermediate (2.5 µg mL^−1^) and high-intermediate (7.5 µg mL^−1^) concentrations. The relative errors found ranged from −13% to +16%. It was therefore concluded that the accuracy was satisfactory according to the standards set for this kind of samples [21]. The precision was evaluated by calculating the relative standard deviations (RSDs) of the areas measured in three consecutive injections (intra-day RSD) and in three different working sessions (inter-day RSDs); both parameters were determined at two different concentrations levels. Although for α-amyrin the RSDs were slightly higher, values <8% were found. Finally, the LODs and LOQs were established. Although different options are available, in this study the LODs and LOQs were calculated as the concentrations that resulted in signal-to-noise ratios of 3 and 10, respectively [22]. These values were established by injecting solutions with decreasing concentrations of the analytes; before analyzing each solution, water was processed to confirm the absence of contaminants and/or memory effects. The LODs were 0.1 µg mL^−1^ for limonene and 0.25 µg mL^−1^ for the rest of compounds; the LOQs were 0.4 µg mL^−1^ for limonene and 0.8 µg mL^−1^ for the other analytes.

### 2.3. Analysis of Resins

#### 2.3.1. Sample Preparation

The proposed conditions were applied to the analysis, to the target compounds in three resins, white copal, copal in tears and resin obtained from ocote trees. Different solubility studies were carried out by treating portions of 1–15 mg of the three resins with 1 mL of extracting solvent. Methanol, acetonitrile, ethyl acetate, isopropanol, and chloroform were tested as extraction solvents.

**Figure 3:**
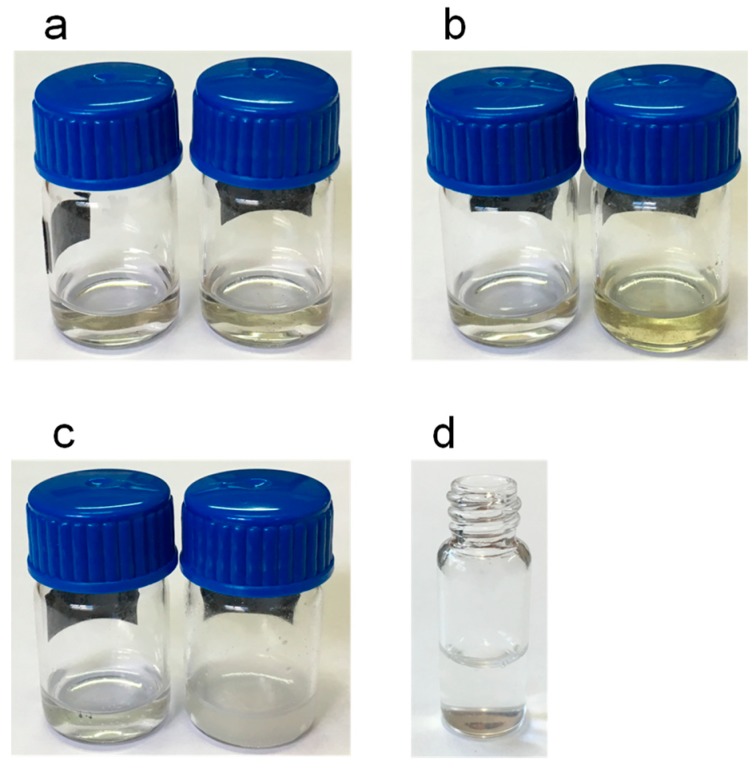
Images of the extracts obtained after adding 1 mL of methanol: (**a**) white copal, (**b**) ocote and (**c**) copal in tears; left vials in (**a**–**c**), 1 mg of samples; right vials in (**a**–**c**), 15 mg of the samples. (**d**) solution obtained after treating the residue insoluble in methanol of copal in tears (10 mg) with 1 mL of water. For other experimental details, see the text.

The chromatograms of the extracts obtained with ethyl acetate, isopropanol and chloroform were unsuitable due to the absorption of these solvents at 200 nm. It was concluded that the employment of such solvents would require the evaporation of the extracts followed by their redissolution in methanol or acetonitrile before the chromatographic analysis. In order to simplify the entire analytical process and to prevent possible losses of the volatile analyte limonene, these solvents were not used in further experiments. Examples of the extracts obtained with methanol are shown in Figure 3. As observed, the white copal (Figure 3a) and ocote (Figure 3b) samples were satisfactorily dissolved. However, significant amounts of solid matter were observed when 10–15 mg of the copal in tears resin was treated with 1 mL of methanol (Figure 3c), most probably due to the presence of highly polar gum compounds [8]. For the latter sample, a further study of the solid residue was carried out after centrifugation and separation of the liquid phase. The residue was treated with 1 mL of water, and complete dissolution was observed (Figure 3d), which confirmed the presence of a high percentage of gum in this sample. Therefore, it was concluded that the target compounds were satisfactorily extracted in methanol. As no significant differences between the chromatograms obtained with methanol and acetonitrile were observed, methanol was finally selected.

The effect of the sample matrix in the response was studied by spiking with known amounts of the analytes the extracts obtained from one of the resin samples (copal in tears) so that the concentration added of each of the analytes to extracts was 5 µg mL^−1^. The increment on the peak areas between the spiked and unspiked extracts was used to calculate the added concentration, using the calibration equations of Table 1. The values obtained were then compared with the added concentrations (5 µg mL^−1^) to calculate the recoveries. Values ranging from 52% to 103% were found, as listed in Table 2.

The minimum percentages of the analytes that could be measured were calculated for samples of 10 mg, taking into account the LOQs of Table 1 and the recoveries of Table 2. The values obtained ranged from 0.004% for limonene to 0.02% for β-amyrin. These values were considered low enough for most applications, making unnecessary extra pre-concentration operations.

#### 2.3.2. Quantification Studies

Finally, the proposed method was applied to the quantitative analysis of the three resins tested. For this purpose, different portions of the samples ranging from 1 to 15 mg were analyzed under the conditions described above. The presence of the analytes in the samples was evaluated from the concordance between the retention times and UV spectra of the suspected peaks and those observed for the standard solutions. Additionally, the presence of a compound was confirmed by fortifying the extracts with standard solutions of such compound.

The only analyte found in the three resins analyzed was α-amyrin. Limonene was found in the white copal and ocote resins, whereas lupeol and β-amyrin were found in the copal in tears sample. As expected, besides the peaks of some of the analytes, peaks of unknown compounds were observed in the samples, particularly at retention times close to that of limonene. However, they could be easily differentiated from this compound through their respective UV spectra. The impurities found in the standard solutions of β-amyrin were not identified in the samples.

The percentages of each of the analytes found in the samples were established from the peak areas and the calibration equations of Table 1 and taking into account the recoveries of Table 2. The results are summarized in Table 3. As deduced from this table, the percentages of the triterpenic compounds were <1%.

**Figure 4:**
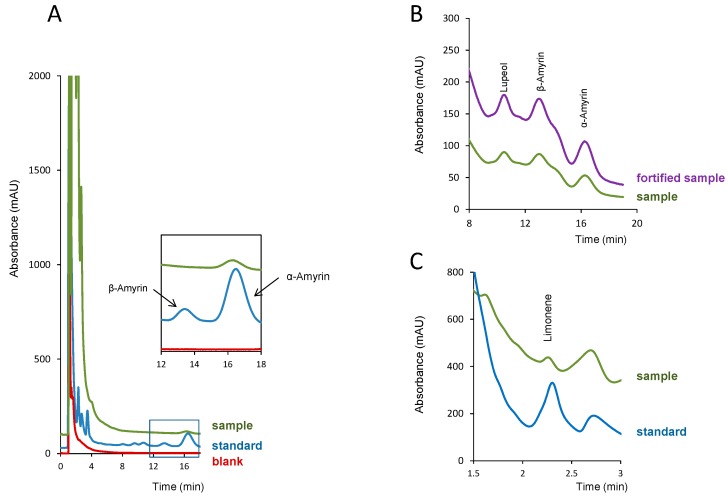
Chromatograms obtained in the analysis of the resin samples: (**a**) white copal, a standard solution of the analytes and a blank (methanol); (**b**) copal in tears (10 mg) and the same extract fortified with the analytes; (**c**) ocote resin diluted 1:20 with methanol and a standard solution of the analytes. Concentration of the analytes in the standard solution of (**a**) and (**c**), 5 µg mL^−1^; amount of the analytes added in the fortified sample of (**b**), 5 µg mL^−1^. For other experimental details, see the text.

For the quantification of these compounds, a higher amount of sample was used (10–15 mg). For 1 mg of the sample, the concentration of α-amyrin in the white copal resin was below its LOD, and between its LOD and LOQ in the ocote resin sample. In the later resin, and even when processing 10 mg of the sample, the concentration of α-amyrin in the extract was close to its LOQ. Limonene was found in white copal and ocote resins at higher percentages. In fact, for the quantification of this analyte in the ocote resin, the extract of the sample had to be diluted with methanol (1:20, *v*/*v*) in order to adjust the analyte concentration to the linear working interval of Table 1. In Figure 4, representative chromatograms obtained for white copal are shown (Figure 4a), copal in tears (Figure 4b) and ocote (Figure 4c) resins; some of the pictures have been zoomed for better visualization of the peaks of interest.

It has to be noted that, because of its relative abundance in the samples, white copal and ocote (≥1%), the percentage of limonene could be established using both 1 mg and 10–15 mg of the samples. The values obtained by using different amounts of the samples were then compared. The *t_calculated_* were 2.01 and 0.17 for white copal and ocote resins, respectively (*t_tabulated_* at 95% confidence level = 2.776); in this calculation, equivalent variances were assumed, as *F_calculated_* were 1.15 and 4.86 for the white copal and ocote resins, respectively (*F_tabulated_* at 95% confidence level = 19.00). Therefore, it was concluded that the percentages obtained were not dependent on the sample size.

Finally, portions of two samples with different composition profiles, copal in tears and ocote, were subjected to different treatments in order to evaluate their effect on the sample composition. For this purpose, portions of the samples were spread on the surface of glass vials; then the vials were exposed at ambient conditions for five days before analysis. Additionally, portions of the samples were dried at 40 °C in an oven until constant weight and then processed. The results obtained are also listed in Table 3. As observed, the composition of the copal in tears sample was not significantly modified by any of the treatments applied. In contrast, both treatments led to lower contents of limonene in the ocote resin, whereas the percentage of α-amyrin increased. The results found for this sample indicate that limonene was partially volatilized both at ambient conditions and after drying at 40 °C; the loss of limonene, and possibly other volatile compounds, resulted in higher percentages of non-volatile compounds such α-amyrin. In the copal in tears resin, the absence of limonene suggests that volatile compounds had been previously lost, which is consistent with the fact that the percentages of the triterpenes remained approximately constant after exposing the sample at ambient conditions or after the thermal treatment applied.

## 3. Discussion

To date, several methods have been proposed for the classification of resins based on the comparison of the fingerprint profiles obtained by chromatographic [7,8,9,10] or spectroscopic techniques [23]. However, few data are available on the quantitative composition of this kind of samples [1,13]. Besides, most of the efforts have been focused on the triterpenoid fraction, while only a few studies have been focused on volatile compounds such as limonene even though this compound can play an important role in establishing the sample botanical origin and age [6].

In this work, we have developed a method for the chromatographic separation, identification, and quantification of compounds commonly found in resins, including the volatile monoterpene limonene and some long-chain non-volatile triterpenes in a single run. Despite the wide range of polarities of the target compounds, satisfactory separation in chromatographic times lower than 20 min was achieved and even under isocratic conditions, which is an additional advantage. As regards its analytical performance, the proposed method provides linear responses, RSDs of 0.6% to 17%, and adequate accuracy [22]. Moreover, because of the high sensitivity attainable with capillary LC, the method is compatible with the analysis of a low amount of the samples. As only a few mg of the samples is necessary, the sample treatment is very simple and avoids heating, multiple extractions or evaporation operations that could modify the content of volatile compound limonene [7,12].

Although significant fluctuations in the levels of the compounds tested can be expected in resins [1], according to the results obtained in our study (Table 3), the method can be applied to the accurate measurement of the analytes at percentages >1% using an amount of sample as low as 1 mg. If the amount of sample available is ≈10–15 mg, compounds present at percentages <0.1% can be also quantified. It is also remarkable that statistically equivalent percentages of the analytes are obtained regardless of the amount of sample (1–15 mg) provided that their concentrations in the corresponding extracts are above their respective LOQs. The percentages of triterpenic compounds found in this study are lower than those reported in other studies, although only few data are available [1]. Variations in the composition of resins can be mostly explained by the high number of species that are used in their production [9,10], although the age and the storage conditions of the samples can also be important sources of variability [6,13]. The proposed method could be used to obtain information relative to the evolution of the chemical composition of resins as a function of the external conditions. In this respect, systematic studies with resins of different botanical origin and age would be necessary. The method should be also tested for other terpenoids.

## 4. Materials and Methods

### 4.1. Chemicals and Solutions

All reagents were of analytical grade. Limonene, lupenone, β-amyrin and α-amyrin standards were obtained from Sigma-Aldrich (St. Louis, MO, USA), and lupeol from Cayman Chemical (Ann Arbor, MI, USA). Methanol and acetonitrile, both HPLC grade, were purchased from VWR Chemicals (Randnor, PA, USA). Ethyl acetate and chloroform super purity solvent were purchased from Romil (Cambridge, UK), and tetrahydrofuran (GPC grade) and isopropanol (HPLC grade) from Scharlau (Barcelona, Spain). Ultrapure water was obtained from an Adrona system (Riga, Latvia). Water was filtered through 0.22 µm nylon membranes purchased from GVS (Sandfor, ME, USA) before use.

Stock solutions of the analytes (1000 µg mL^−1^) were prepared by dissolving the appropriate amounts of the commercial standards in methanol. Working solutions of the analytes and their mixtures were prepared by diluting the stock solutions with methanol (unless otherwise stated). All solutions were stored at 4 °C until use.

### 4.2. Instrumentation and Analytical Conditions

The chromatographic system consisted of a capillary pump (Agilent 1100 Series, Waldbronn, Germany) equipped with a Rheodyne model 7725 six-port injection valve and a photodiode array detector (Agilent 1200 Series). An Agilent HPLC ChemStation system was used for data acquisition and calculation.

A Zorbax SB C18 (150 mm × 0.5 mm id, 5 µm) column (Agilent) was used for the separation of the target compounds. Unless otherwise stated, the mobile phase was a mixture acetonitrile:water (85:15, *v*/*v*) at a flow rate of 10 µL min^−1^. A 15-cm segment of 0.320 mm o.d. and 75 µm i.d. fused silica capillary (Análisis Vínicos, Tomelloso, Spain) was used as the injection loop; for connecting the capillary to the valve, 2.5-cm sleeves of 1/6 in polyether ether ketone (PEEK) tubing (1/6 in PEEK nuts and ferrules) from Teknokroma (Barcelona, Spain) were used. Working solutions were loaded into the loop employing a 25 µL precision syringe. The analytical signal was recorded between 190 and 400 nm and monitored at 200 nm.

### 4.3. Analysis of Resins

Samples of different commercial resins were analyzed, white copal and copal in tears, as well as a resin obtained from ocote trees. Samples were purchased in Sonora market (City of México, México) in the year 2010. Portions of the resins were homogenized mechanically in a mortar with a pestle. Next, accurately weighted portions of the pulverized samples (≈1–15 mg) were placed in 2 mL glass vials and treated with 1 mL of extraction solvent. Acetonitrile, methanol, chloroform, isopropanol, and ethyl acetate were tested as extraction solvents. The mixture was vortexed for 1 min and then filtered through 0.22 µm nylon membranes to remove any particulate that could be present. Finally, aliquots of 5 µL of the samples were chromatographed. All the experiments were carried out at room temperature by triplicate.

## 5. Conclusions

In this work, we have developed a method for the quantitative analysis of some relevant terpenoids typically used to characterize of resins, which is based on capillary LC with UV detection. Separation and chromatographic conditions have been optimized to make possible the analysis of volatile and non-volatile analytes within the same chromatographic run, with the adequate sensitivity to be applied when only small size samples are available (a few mg).

The results obtained throughout our study have proved that the quantitative performance of the proposed method is suitable. To the best of our knowledge, this is the first method validated for the quantification of limonene and representative triterpenes in microsamples of resins. Thus, it can be considered a useful tool to increase the knowledge about the chemical composition of resins, as most existing methods are limited to obtain their chemical fingerprints. Besides for classification purposes, the quantitative composition can be used to obtain information about the history (age and ambient conditions) of samples of similar origin.

## Figures and Tables

**Table 1:** Analytical parameters of the proposed method.

Compound	Linearity *, ** (*n* = 15)		Mean Found Concentration ** (*n* = 3)		Precision, rsd (%) (*n* = 3)				LOD (µg mL^−1^)	LOQ (µg mL^−1^)
	y = (a ± s_a_) + (b ± s_b_) x	R^2^	2.5 µg mL^−1^	7.5 µg mL^−1^	Intraday		Interday			
					2.5 µg mL^−1^	7.5 µg mL^−1^	2.5 µg mL^−1^	7.5 µg mL^−1^		
Limonene	y = (−77 ± 2) + (433 ± 6)x	0.997	2.3 ± 0.1	6.5 ± 0.4	2	0.6	3	4	0.1	0.4
Lupenone	y = (−22 ± 9) + (63.9 ± 1.6)x	0.994	2.4 ± 0.1	7.0 ± 0.1	4	0.8	7	7	0.25	0.8
Lupeol	y = (−42 ± 12) + (111 ± 2)x	0.996	2.6 ± 0.1	7.4 ± 0.2	1.4	2	7	8	0.25	0.8
β-Amyrin	y = (−20 ± 17) + (135 ± 3)x	0.995	2.3 ± 0.1	7.5 ± 0.6	3	8	8	8	0.25	0.8
α-Amyrin	y = (72 ± 42) + (313 ± 8)x	0.994	2.9 ± 0.2	8.4 ± 0.1	9	17	16	17	0.25	0.8

* within the range 0.25–10.0 μg mL^−1^ for limonene and 0.5–10.0 μg mL^−1^ for the rest of compounds (*a*: intercept; *s_a_*: standard deviation of the intercept; *b*: slope; *s_b_*: standard deviation of the slope; *R^2^*: squared correlation coefficient; *rsd*: residual standard deviation); ** all values expressed with digits known plus the first uncertain digit.

**Table 2:** Recoveries * obtained from the spiked extracts (*n* = 3).

Compound	Recovery (%)
Limonene	103 ± 4
Lupenone	101 ± 1
Lupeol	79 ± 9
β-Amyrin	52 ± 5
α-Amyrin	75 ± 3

(*) All values expressed with digits known plus the first uncertain digit.

**Table 3:** Percentages * of the analytes found in the analyzed resin samples (*n* = 3).

Sample		Percentage ^a^ (%), (*n* = 3)				
		Limonene	Lupenone	Lupeol	β-Amyrin	α-Amyrin
**White copal**	1 mg	0.9 ± 0.2	<LOD	<LOD	<LOD	<LOD
	15 mg	1.2 ± 0.2	<LOD	<LOD	<LOD	0.020 ± 0.002
**Copal in tears**	10 mg	<LOD	<LOD	0.034 ± 0.001	0.069 ± 0.002	0.011 ± 0.001
	10 mg ^b^	<LOD	<LOD	0.033 ± 0.001	0.074 ± 0.001	0.010 ± 0.003
	10 mg ^c^	<LOD	<LOD	0.035 ± 0.002	0.082 ± 0.005	0.010 ± 0.004
**Ocote**	1 mg	9.3 ± 0.2	<LOD	<LOD	<LOD	<LOQ
	10 mg	9.3 ± 0.1	<LOD	<LOD	<LOD	0.093 ± 0.003
	10 mg ^b^	7.2 ± 0.1	<LOD	<LOD	<LOD	0.16 ± 0.01
	10 mg ^c^	7.3 ± 0.3	<LOD	<LOD	<LOD	0.16 ± 0.02

(^a^) All values expressed with digits known plus the first uncertain digit; (^b^) Exposed at ambient conditions for 5 days; (^c^) Dried at 40 °C until constant weight.
